# Anticancer Effects of High Glucosinolate Synthesis Lines of *Brassica rapa* on Colorectal Cancer Cells

**DOI:** 10.3390/antiox11122463

**Published:** 2022-12-14

**Authors:** Jung Sun Kim, Sanghee Han, Hail Kim, So Youn Won, Hyun Woo Park, Hyunjin Choi, Minji Choi, Min Young Lee, In Jin Ha, Seok-Geun Lee

**Affiliations:** 1 Genomic Division, Department of Agricultural Bio-Resources, National Institute of Agricultural Sciences, Rural Development Administration, Jeonju 54874, Republic of Korea; 2 Graduate School, Kyung Hee University, Seoul 02447, Republic of Korea

**Keywords:** *Brassica rapa*, glucosinolate, hybridization, doubled haploid lines, chemoprevention, colorectal cancer

## Abstract

Chemoprevention is a method of health control in modern industrialized societies. Traditional breeding (hybridization) has been widely used to produce new (sub)species with beneficial phenotypes. Previously, we produced a number of doubled haploid (DH) lines of *Brassica rapa* with a high glucosinolate (GSL) content. In this study, we evaluated the anticancer activities of extracts from three selected high-GSL (HGSL)-containing DH lines (DHLs) of *Brassica rapa* in human colorectal cancer (CRC) cells. The three HGSL DHL extracts showed anti-proliferative activities in the 3-(4,5-dimethylthiazol-2-yl)-2,5-diphenyltetrazolium assay and pro-apoptotic activities in the cell cycle or annexin V analysis with the induction of pro-apoptotic protein expression in CRC cells. Mechanistically, HGSL DHL extracts inhibited the NF-κB and ERK pathways, leading to a reduction in the nuclear localization of NF-κB p65. In addition, reactive oxygen species were induced by HGSL DHL extract treatment in CRC cells. In conclusion, our data suggest that the newly developed HGSL DHLs possess enhanced anticancer activities and are potentially helpful as a daily vegetable supplement with chemopreventive activities.

## 1. Introduction

Colorectal cancer (CRC) makes up approximately 10% of all annually diagnosed cancers and was responsible for 9.4% of all cancer-related deaths worldwide in 2020 [1]. It is the second most common cancer diagnosed in women, the third most common cancer in men, and the second deadliest cancer [1]. The incidence rates of CRC are approximately 4-fold higher in developed countries than in transitioning countries, but the disparity in its mortality rate is somewhat less because of the higher fatality in transitioning countries [1]. CRC can be pondered as a marker of socioeconomic development, and in countries under the main transition, incidence rates are apt to rise constantly with the human development index (HDI) [1,2]. The elevation in formerly low-risk and lower HDI countries likely mirrors changes in lifestyle; shifts toward increased consumption of meats and a more inactive lifestyle, causing reduced physical activity and rising prevalence of overweight and obesity, which are independently associated with CRC risk [1,2,3]. Heavy alcohol consumption and cigarette smoking are additional risk factors, but calcium supplements and the adequate consumption of whole grains, fiber, and dairy products must be good to decrease the CRC risk [1,2,3]. Moreover, inflammation is also involved in CRC development and progression, and individuals with long-standing ulcerative colitis and inflammatory bowel disease require adequate surveillance [2,4].

Edible plants are a well-established source of phytochemicals, including herbal medicines [5]. Accumulating data supports phytochemicals from green vegetables as key components in promoting human health and longevity with antioxidant and anti-inflammatory activities. Although molecular genetic engineering is now the most advanced technology, the hybridization (breeding) of (sub)species remains a valuable tool for the development of new species with desired strengthened phenotypes and/or genotypes [6,7]. Modern genomic techniques, such as whole genome sequencing, resequencing, and pan-genome analysis, now support the molecular basis of new species with selected phenotypes [6].

*Brassica rapa* is widely consumed in East Asian cuisine [8]. Glucosinolates (GSLs) are a group of organic compounds with highly diverse structures in plants [9] and are among the major defense substances found in the Brassicaceae family [10]. Myrosinase-mediated hydrolysis of GSLs produces bioactive compounds, including thiocyanates, isothiocyanates (ITCs), and nitriles in cruciferous vegetables that have anticancer activity in mammalian cells [10,11]. ITCs, such as benzyl isothiocyanate (BITC), phenyl isothiocyanate (PEITC), and 1-isothioyanato-4-(methyl-sulfinyl) butane (sulforaphane [SFN]), stimulate reactive oxygen species (ROS), cell cycle arrest, apoptosis, and autophagy; therefore, they have been established as potent anticancer and chemopreventive agents against various types of cancer [11,12]. In addition, the hydrolyzed products of GSLs have been reported to induce the activity of nuclear factor-erythroid-2-related factor 2 (NRF2), a well-known master transcriptional regulator of a variety of genes involved in the oxidative stress response, detoxification, and drug resistance [13,14,15,16,17]. The activation of NRF2 leads to the inhibition of the activity of nuclear factor kappa B (NF-κB) through antioxidant enzymes, such as heme oxygenase (HO-1), and the blocking of the proteasomal degradation of NF-κB inhibitor-alpha (IκBα) [11,18,19].

Previously, we generated a series of doubled haploid (DH) lines (DHLs) from two different edible parental *Brassica rapa* subspecies: *B. rapa* subsp. *trilocularis* (yellow sarson) and *B. rapa* subsp. *chinensis* (pak choi (PC)) [20]. From 161 DHLs, we identified high-GSL (HGSL)-containing DHLs with GSL contents ranging from 44.12 to 57.04 μmol/g·dry weight (dw). In this study, we evaluated the anticancer effects of crude extracts from three selected edible HGSL DHLs (DH005, DH014, and DH026) compared to those of PC in human CRC cells.

## 2. Materials and Methods

### 2.1. Plant Materials and Sample Preparation

Previously, we generated a series of DHLs from two different edible parental plants, *B. rapa* subsp. *trilocularis* (yellow sarson) and *B. rapa* subsp. *Chinensis* (pak choi) [20]. We used three HGSL DHLs with BioSample accession numbers deposited in the NCBI database as follows: BrYSP_DH005, BrYSP_DH014, and BrYSP_DH026, which were deposited in Korea Agricultural Culture Collection (KACC, http://genbank.rda.go.kr, accessed on 16 September 2020) as follows: BrYSP_DH005 (KACC98082P), BrYSP_DH014 (KACC98083P), and BrYSP_DH026 (KACC98086P). The sample preparations for the current study were always confirmed by verification of GSL contents. For analysis of GSL contents in the samples, desulfated GSL was quantified in Agilent 1200 Series high-performance liquid chromatography (HPLC) System (Agilent Technologies, Santa Clara, CA, USA) equipped with an Inertsil ODS-3 column (150 × 3.0 mm inner diameter, particle size 3 µm; GL Science, Tokyo, Japan). Analysis was performed using a flow rate of 0.4 mL·min^−1^ at a column over 35 °C and a wavelength of 227 nm. Sinigrin was used as an external standard for quantification. Total and individual GSL contents were calculated as means of three biological replicates [20]. Samples of commercial *B. rapa* subsp. *chinensis* (PC) and the three selected HGSL DHLs were immediately frozen in liquid nitrogen and freeze-dried (FDU-2110, EYELA, Tokyo, Japan). For extract preparation, freeze-dried sample powder (500 mg) was extracted with 25 mL of RPMI 1640 medium (10-040-CV, Mediatech Inc. a Corning subsidiary, Manassas, VA, USA) at room temperature and sonicated for 1 h. Each extract was filtered using a Minisart^®^ 0.45-µm syringe filter (Sartorius, Göttingen, Germany).

### 2.2. Cell Lines and Reagents

Two CRC cell lines, HCT116 and HT29, and one Dukes’ type B colorectal adenocarcinoma cell line SW480 were purchased from the Korean Cell Line Bank (Seoul, Republic of Korea) and maintained in Roswell Park Memorial Institute (RPMI) 1640 medium containing 10% fetal bovine serum according to the manufacturer’s recommendations. The number of cells was monitored by trypan blue dye exclusion assay using either a manual hemocytometer or an automated cell counter [21].

### 2.3. Cell Viability Assay

An appropriate number of cells (5–20 × 10^3^ cells/well) were seeded into 96-well plates. One day after seeding, the cells were treated with normal growth media containing the indicated amounts of extracts for 48 or 72 h. Cell viability was determined by the 3-(4,5-dimethylthiazol-2-yl)-2,5-diphenyltetrazolium (MTT) assay, as described previously [22].

### 2.4. Invasion Analysis

Boyden chamber membranes (Neuro Probe; Cabin John, MD, USA) were coated with Matrigel (Corning, Corning, NY, USA) for 1 h, and cells (4–8 × 10^4^ cells/well) were seeded onto the coated Matrigel. Cells were treated with the extracts for 24 h and visualized using Giemsa staining. The number of invaded cells was determined by microscopic observation.

### 2.5. Cell Cycle and Apoptosis Analysis

Cells were seeded in 6-well plates at a density of 2–3 × 10^5^ cells/well and treated with the extract for 48 h. For cell cycle analysis, the cells were stained with propidium iodide (PI) after ethanol fixation. To analyze apoptotic cells, the cells were stained with annexin V-fluorescein isothiocyanate (FITC)-PI. The distribution of each cell in the cell cycle and apoptotic cells was analyzed using a flow cytometer (BD FACSCanto^TM^ II; BD Biosciences, Franklin Lakes, NJ, USA) as described previously [23]. A terminal deoxynucleotidyl transferase dUTP nick end labeling (TUNEL) assay was also performed as described previously [23].

### 2.6. Western Blot Analysis

Whole-cell lysates were prepared, and Western blotting was performed as previously described [23]. Primary antibodies against phospho-p44/42 MAPK (p-ERK1/2) (Thr202/Tyr204), ERK1/2, p-IκBα (Ser32/36), IκBα, p-p65 (Ser536), p65, and COX2 were purchased from Cell Signaling Technology (Danvers, MA, USA). Additionally, antibodies for Bcl-2, Bax, caspase-3, cleaved-caspase-3, and β-actin were purchased from Santa Cruz Biotechnology (Santa Cruz, CA, USA), and HRP-conjugated anti-mouse IgG and anti-rabbit (Cell Signaling Technology) were used. Densitometric analysis of each band was performed using ImageJ software (https://imagej.nih.gov/ij, accessed and downloaded the updated software on 27 September 2021). Data are presented as the mean ± standard deviation (SD) from at least three independent experiments.

### 2.7. Analysis of Subcellular Localization of p65

Immunostaining for phospho (p)-p65 was performed as described previously [23]. Images were obtained using an iRiS^TM^ Digital Cell Imaging System (Logos Biosystems, Anyang, Republic of Korea).

### 2.8. Analysis of Reactive Oxygen Species (ROS) Generation

Intracellular ROS production was measured using 2,7-dichlorofluorescein diacetate (DCF-DA) as previously described [24]. The cells were incubated with each extract for 24 h and stained with 20 µM DCF-DA (#D6883; Sigma-Aldrich, St. Louis, MO, USA) for 30 min at 37 °C in the dark. They were then collected, washed twice with PBS, and analyzed a total of 10,000 events using the flow cytometer (BD FACSCanto^TM^ II).

## 3. Results

### 3.1. Anti-Proliferation Effects of HGSL DHL Extracts in Human CRC Cells

**Figure 1:**
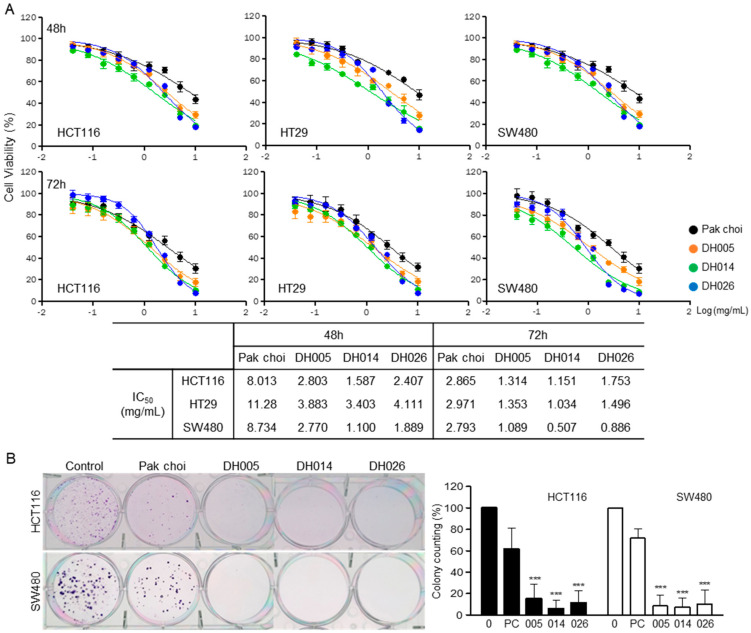
Anti-proliferation effects of high glucosinolate (GSL) (HGSL) doubled haploid (DH) lines (DHLs) (DH005, DH014, and DH026) in human colorectal cancer (CRC) cells. (**A**) HCT116, HT29, and SW480 cells were treated with each HGSL DHL extract or pak choi (PC) extract as a parental control for 48 or 72 h. Cell viability was measured by 3-(4,5-dimethylthiazol-2-yl)-2,5-diphenyltetrazolium (MTT) assays and each half-maximal inhibitory concentration (IC_50_) value of every human CRC cell line was determined. Data in the graphs are presented as the mean ± standard error of the mean (SEM). (**B**) Colony formation assays in monolayer culture were performed in HCT116 and SW480 cells treated with 1.25 mg/mL of each sample for 2 weeks. Colonies were fixed, stained, and counted (>50 cells). Data represent the mean ± standard deviation (SD) of three independent experiments (***, *p* < 0.001 versus control).

The effects of each HGSL DHL extract on the proliferation of CRC cells were determined and compared to those of PC extract using the MTT cell viability assay. Cells cultured in 96-well plates were treated with normal growth medium containing crude extracts of HGSL DHLs or PC for 48 and 72 h. The cell viability and half-maximal inhibitory concentration (IC_50_) were determined, as shown in Figure 1A. All extracts showed enhanced anti-proliferative effects against the three CRC cell lines compared to the control extract from PC. The antiproliferative effects of HGSL DHL extracts were dose- and time-dependent. DH014 had the most profound effect on CRC cells after 48 and 72 h of treatment.

The effects of each extract on the long-term clonogenic potential of the CRC cells were also evaluated. CRC cells were treated with 1.25 mg/mL of extracts for 2 weeks, and surviving colonies were visualized and counted. The selected concentration of extracts was around the mean IC_50_ values of all extracts from the MTT assay. All HGSL DHL extracts showed significant anti-clonogenic effects in CRC cells compared with those shown by the PC control (Figure 1B).

### 3.2. Effects of HGSL DHL Extracts on the Invasion of Human CRC Cells

**Figure 2:**
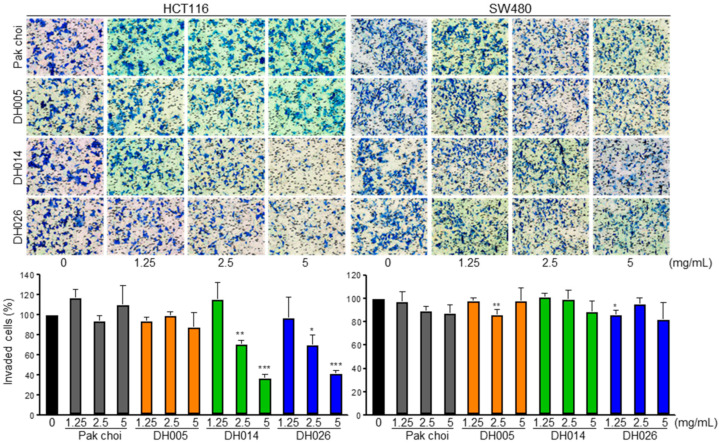
Anti-invasion effects of high glucosinolate (GSL) (HGSL) doubled haploid (DH) lines (DHLs) (DH005, DH014, and DH026) in human colorectal cancer (CRC) cells. HCT116 and SW480 cells in the serum-free media were seeded onto the upper side of Boyden chambers, treated with either HGSL DH or pak choi extract, and 24 h later the membranes were fixed, stained, and photographed. Invaded cells were counted in three randomly chosen fields of each membrane. Data in the bar graphs represent the mean ± SEM of three independent experiments in duplicate or triplicate (*, *p* < 0.05; **, *p* < 0.01 and ***, *p* < 0.001 versus control).

The effect of each extract on CRC cell invasion was evaluated using the Boyden chamber assay. Cells cultured in the Matrigel-coated upper chamber were treated with normal growth media containing HGSL DH or PC extract for 24 h, and the number of invaded cells was determined (Figure 2). In contrast, all extracts showed enhanced anti-proliferation effects against the three CRC cell lines compared to the PC extract, and the effects of HGSL DHL extracts on CRC cell invasion were different. DH014 and DH026 exerted a dose-dependent inhibition of cell invasion in HCT116 cells. In contrast, DH005 and DH026 marginally affected SW480 cell invasion. The PC extract did not show any effect on the invasion of either CRC cell line.

### 3.3. Induction of Apoptosis by HGSL DHL Extracts in Human CRC Cells

**Figure 3:**
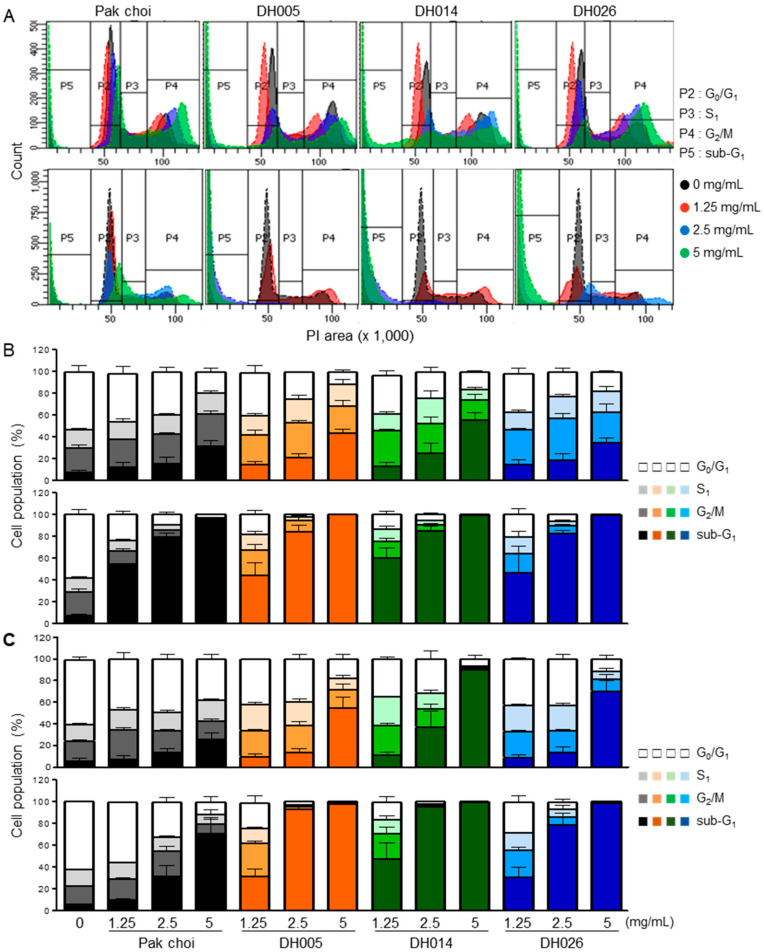
Effects of high glucosinolate (GSL) (HGSL) doubled haploid (DH) lines (DHLs) (DH005, DH014, and DH026) on the cell cycle in human colorectal cancer (CRC) cells. HCT116 and SW480 cells were treated with each HGSL DHL extract or pak choi extract as a control for 24 or 48 h. The treated cells were stained with propidium iodide (PI), and the staining was analyzed using flow cytometry. Every PI staining and analysis was performed at least three times in duplicate. (**A**) The representative analysis of the 24 h-treated cells (upper: HCT116 and lower: SW480) was shown in the histograms. The population of HCT116 (**B**) and SW480 (**C**) cells were quantitated as a percentage of cell numbers in the G_0_/G_1_, S, G_2_/M, and sub-G_1_ phases. Data (upper: 24 h and lower: 48 h) represent the mean ± SEM.

The effects of HGSL DHL extract on cell cycle progression and apoptosis were analyzed by PI and Annexin V staining, respectively. HCT116 and SW480 cells were treated with increasing amounts of HGSL DH or PC extract for 24 or 48 h. As shown in Figure 3, treatment with HGSL DHL extract reduced the G_0_/G_1_ phase of CRC cells in a dose-dependent manner. Concomitantly, the sub-G_1_ phase population was markedly induced by HGSL DHL extracts. Compared to the PC control, near-complete abrogation of the cell cycle was observed in CRC cells treated with 2.5 mg/mL of HGSL DHL extracts after 48 h (Figure 3B,C).

**Figure 4:**
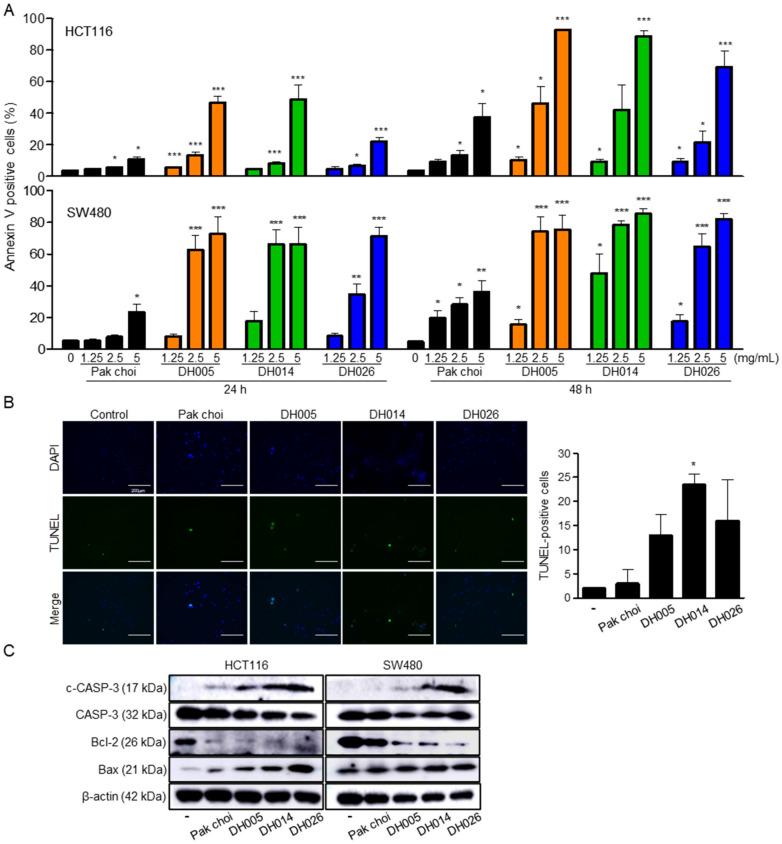
Effects of high glucosinolate (GSL) (HGSL) doubled haploid (DH) lines (DHLs) (DH005, DH014, and DH026) on apoptosis in human colorectal cancer (CRC) cells. (**A**) HCT116 and SW480 cells were treated with each HGSL DH or pak choi (PC) extract for 24 or 48 h. The treated cells were double-stained with fluorescein isothiocyanate (FITC)-Annexin V and propidium iodide (PI) and analyzed using flow cytometry. Apoptotic cells were quantitated as a percentage of Annexin V-positive cells. Data represent the mean ± SEM of three independent experiments (*, *p* < 0.05; **, *p* < 0.01 and ***, *p* < 0.001 versus control). (**B**) The 24 h-treated HCT116 cells were stained with terminal deoxynucleotidyl transferase dUTP nick end labeling (TUNEL) and 4′,6-diamidino-2-phenylindole (DAPI) and analyzed by confocal microscopy (scale bar, 200 μm). TUNEL-positive cells were counted on at least three fields in each well, and data represent the mean ± SD (*, *p* < 0.05). (**C**) HCT116 and SW480 cells were treated with each sample (2.5 mg/mL) for 24 h. Then, the whole cell lysates were prepared and subjected to Western blotting with the antibodies against Bcl-2, Bax, caspase-3 (CASP-3), and cleaved-CASP-3 (c-CASP-3). β-actin was used as an internal control.

Since an increase in the sub-G_1_ phase implies the induction of apoptosis, we further determined the effects of HGSL DHL extracts on apoptosis by annexin V staining. Marked increases in annexin V-stained cells were observed even after 24 h of treatment with 5 mg/mL HGSL DHL extracts in CRC cells (Figure 4A). Consistent with the results of proliferation assays and cell cycle analysis, every HGSL DHL extract caused much more apoptotic death of human CRC cells than the PC extract did, and DH014 had the most profound effect on the induction of apoptosis.

Apoptotic cells were further visualized and analyzed using the TUNEL assay in HCT116 cells. The most profound induction of apoptosis was observed in DH014 extract-treated cells (Figure 4B). Western blot analysis demonstrated an increase in cleaved-caspase-3 and Bax expression levels and an abrogation of anti-apoptotic protein Bcl-2 expression following HGSL DHL extract treatment, further confirming the induction of apoptosis (Figure 4C).

### 3.4. Induction of ROS by HGSL DHL Extracts in Human CRC Cells

**Figure 5:**
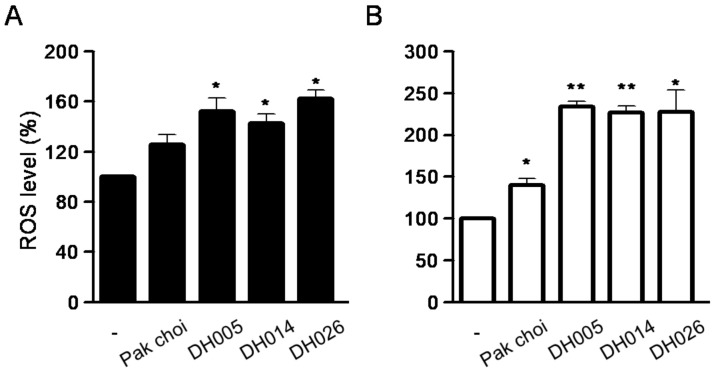
Effects of high glucosinolate (GSL) (HGSL) doubled haploid (DH) lines (DHLs) (DH005, DH014, and DH026) on intracellular reactive oxygen species (ROS) production in human colorectal cancer (CRC) cells. HCT116 (**A**) and SW480 (**B**) cells were treated with 2.5 mg/mL of each HGSL DH or pak choi extract for 24 h. ROS generation was measured by 2,7-dichlorofluorescein diacetate (DCF-DA) staining with flow cytometry analysis. Every staining and analysis was performed at least three times in duplicate. Data represent the mean ± SEM. (*, *p* < 0.05 and **, *p* < 0.01 versus control).

Since ITCs and many anticancer drugs are known to cause intracellular ROS accumulation, leading to apoptotic cell death, we further analyzed the amount of ROS in CRC cells after treatment with HGSL DHL extracts. As shown in Figure 5, all HGSL DHL extracts markedly increased the amount of ROS compared to the treatment of the PC extract.

### 3.5. Inhibition of the NF-κB Signaling Pathway by HGSL DHL Extracts in Human CRC Cells

**Figure 6:**
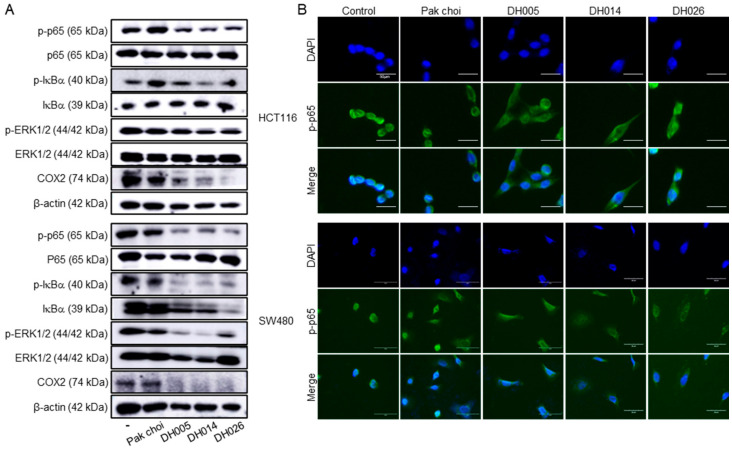
Effects of high glucosinolate (GSL) (HGSL) doubled haploid (DH) lines (DHLs) (DH005, DH014, and DH026) on the NF-κB pathway in human colorectal cancer (CRC) cells. HCT116 and SW480 cells were treated with 2.5 mg/mL of each HGSL DH or pak choi (PC) extract for 24 h. (**A**) Whole-cell lysates were prepared and subjected to Western blotting for phospho (p)-p44/42 MAPK (ERK1/2) (Thr202/Tyr204) (p-ERK1/2), ERK1/2, p-IκBα (Ser32/36) (p-IκBα), IκBα, p-p65 (Ser536) (p-p65), p65, and COX2. β-actin was used as an internal control. (**B**) The treated cells were subjected to double immunofluorescence staining against p-p65 and 4′,6-diamidino-2-phenylindole (DAPI) staining and analyzed by confocal microscopy (scale bar, 50 μm).

As CRC is closely related to inflammation and NF-κB plays an essential role in inflammation-associated cancer progression [2,4,25], we investigated the effects of HGSL DHL extracts on the NF-κB pathway in CRC cells. CRC cells were treated with 2.5 mg/mL of HGSL DH or PC extracts for 24 h, and the whole-cell lysates were analyzed by Western blot (Figure 6A). Interestingly, all HGSL DHL extracts reduced the phosphorylation level of p65, whereas the PC extract showed little or no effect on CRC cells. Consistently, IκBα phosphorylation was also reduced by all HGSL DHL extracts. Notably, the reduction in total IκBα levels was only distinctly observed in SW480 cells under these conditions. Since the regulation of p65 and IκBα phosphorylation results in a change in NF-κB subcellular localization and activity, we further analyzed the effects of HGSL DHL extracts on the subcellular localization of p65. As shown in Figure 6B, treatment with HGSL DHL extracts abolished the nuclear localization of p65 and induced cytoplasmic accumulation of p65 compared to the negative control and PC-treated cells. In addition, the level of COX2, an NF-κB target, was reduced by all HGSL DHL extracts, except for the PC extract. Since NF-κB cooperates with many other signaling molecules for inflammation-associated tumorigenesis [25], we also examined the effect of HGSL DHL extracts on the phosphorylation of ERK1/2, which is a key molecule usually associated with cell proliferation and growth in the MAPK pathway, one of the signaling pathways that crosstalk with NF-κB in cancer cells. As shown in Figure 6A, all HGSL DHL extracts decreased the phosphorylation level of ERK1/2 but had no effect on the phosphorylation of p65. Taken together, these results suggest that HGSL DHL extracts inhibit the NF-κB and ERK pathways and the transcriptional activation of NF-κB by reducing the nuclear localization of p65 in CRC cells.

## 4. Discussion

Dietary lifestyles containing high amounts of vegetables and fruits play a crucial role in preventing cancer or proliferative diseases [26]. To produce new species with enriched GSL contents, we previously generated intercrossed subspecies of *B. rapa* subsp. *trilocularis* and *B. rapa* subsp. *chinensis* using microspore culture [20]. Resequencing and GSL analysis revealed that the total GSL contents of newly produced HGSL DHLs ranged from 44.12–57.04 μmol/g. DHLs with high GSL amounts were characterized as containing biosynthesis-specific recombinant blocks in their genome, which encode multiple GSL biosynthetic genes, such as branched-chain amino acid aminotransferase 4 (BCAT4), methylthioalkylmalate synthase 1 (MAM1), MAM3, bile acid transporter 5 (BAT5), 2-oxoglutarate-dependent dioxygenase 1 (AOP1), AOP2, and APS kinase 1 (APK1). As a result, HGSL DHLs, such as DH005, DH014, and DH026, showed >5-fold increases in GSL hydrolysis products, including BITC, PEITC, and SFN, compared to those in the commercial PC cultivar control. In this study, we characterized the anticancer effects of HGSL DHLs in human CRC cells.

GSL biosynthesis in *Brassica* species is regulated by myeloblastosis (MYB) transcription factors [27,28,29,30]. Among the 63 orthologous copies found in *Brassica* species, 55 MYB transcription factors have a conserved amino acid sequence in their R2R3 DNA-binding domain. Recently, GSLs have been reported as enriched secondary metabolites, with more than 120 different GSLs in Brassicaceae [31,32]. Although GSLs themselves are not bioactive, the hydrolysis of GSLs by endogenous myrosinase produces bioactive chemopreventive compounds, including ITCs, nitriles, and indoles [33]. ITCs, including BITC, PEITC, and SFN, are well-known chemopreventive and anticancer agents against multiple types of cancer [11,12]. The HGSL DHLs we selected (DH005, DH014, and DH026) showed >5-fold increases in GSL-hydrolyzed products. Among them, DH014 had the highest number of ITCs, with approximately 7.2-, 3.0-, 3.1-, and 3.5-fold higher BITC, 4-PEITC, 2-PEITC, and SFN, respectively, compared to that in the PC control. As expected, the highest anti-proliferative activity was observed in CRC cells treated with DH014 extract (Figure 1A). Consistently, the long-term survival of CRC cells was also inhibited by DH extracts with the highest activity of DH014 (Figure 1B). The DH014 extract also showed profound effects on HCT116 cell migration (Figure 2) and apoptotic cell death in HCT116 and SW480 cells (Figure 3 and Figure 4). In addition, the blocking of signaling pathways—including IKK/NF-κB and ERK—by HGSL DHL extracts led to the downregulation of COX2 protein expression, probably via inhibition of the nuclear localization of NF-κB p65 in CRC cells. All HGSL DHL extracts induced ROS production in CRC cells. Taken together, the new HGSL DHLs have enhanced anticancer activities in CRC cells compared to the parental PC.

Although recent advances in anticancer therapeutics have provided multiple treatment options against a variety of human cancers, the incidence of cancers as well as cancer-associated death rates have not improved owing to heavy industrialization [1]. Dietary control, including green vegetables, can supply beneficial phytochemicals that promote human health and longevity [5]. More importantly, the chemopreventive effects of ITCs, the hydrolyzed products of GSLs, have been established to be mediated by either direct or indirect activation of the master antioxidative transcription factor NRF2 [13,14,15,16,17]. Activated NRF2 induces multiple genes involved in oxidative stress, detoxification, and drug resistance responses. NRF2 also inhibits NF-κB via its antioxidant enzymes [11]. Consistent with these results, extracts from HGSL DHLs had anti-proliferative effects in CRC cell lines with an apparent reduction in NF-κB activation, as shown by subcellular localization and Western blot analyses (Figure 6). Collectively, our data suggest that traditional breeding is still a valid technique for producing invaluable new edible plants with fortified chemopreventive activities for use in the daily diet and that the newly developed HGSL DHLs acquire increased anticancer activities and would be helpful as vegetable supplements with chemopreventive activities.

## Data Availability

The data presented in this study are available in the article.
